# *Halomonas alkaliantarctica* as a platform for poly(3‐hydroxybutyrate‐*co*‐3‐hydroxyvalerate) production from biodiesel‐derived glycerol

**DOI:** 10.1111/1758-2229.13225

**Published:** 2023-12-26

**Authors:** Justyna Możejko‐Ciesielska, Krzysztof Moraczewski, Sylwester Czaplicki

**Affiliations:** ^1^ Department of Microbiology and Mycology, Faculty of Biology and Biotechnology University of Warmia and Mazury in Olsztyn Olsztyn Poland; ^2^ Institute of Materials Engineering Kazimierz Wielki University Bydgoszcz Poland; ^3^ Department of Plant Food Chemistry and Processing University of Warmia and Mazury in Olsztyn Olsztyn Poland

## Abstract

Polyhydroxyalkanoates (PHAs) are biodegradable polyesters produced by a wide range of microorganisms, including extremophiles. These unique microorganisms have gained interest in PHA production due to their ability to utilise low‐cost carbon sources under extreme conditions. In this study, *Halomonas alkaliantarctica* was examined with regards to its potential to produce PHAs using crude glycerol from biodiesel industry as the only carbon source. We found that cell dry mass concentration was not dependent on the applying substrate concentration. Furthermore, our data confirmed that the analysed halophile was capable of metabolising crude glycerol into poly(3‐hydroxybutyrate‐*co*‐3‐hydroxyvalerate) copolymer within 24 h of the cultivation without addition of any precursors. Moreover, crude glycerol concentration affects the repeat units content in the purified PHAs copolymers and their thermal properties. Nevertheless, a differential scanning calorimetric and thermogravimetric analysis showed that the analysed biopolyesters have properties suitable for various applications. Overall, this study described a promising approach for the valorisation of crude glycerol as a future strategy of industrial waste management to produce high value microbial biopolymers.

## Graphical abstract

Biodiesel‐derived glycerol as carbon source for polyhydroxyalkanoate (PHA) accumulation in *Halomonas alkaliantarctica*. *Halomonas alkaliantarctica* is a potent halophilic PHA producer. It is capable of producing poly(3‐hydroxybutyrate‐*co*‐3‐hydroxyvalerate) copolymer.

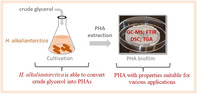

## INTRODUCTION

Industrial biotechnology continues to drive innovation, improve bioprocess efficiency and contribute to a more sustainable and circular bioeconomy. In the last few decades, microbial processes are attracting interests of the scientific community as the sources of value‐added bio‐based products. Among them, polyhydroxyalkanoates (PHAs) are especially attractive as ecological alternative to conventional polymers derived from fossil fuels. They are a group of biopolymers that are produced by certain microorganisms in the form of intracellular granules. For many years, PHAs were thought to be a way to store carbon and energy (Policastro et al., 2021). However, recent studies demonstrated that the biological role of PHAs is more complex. The data suggested that the accumulation of PHAs provide stress robustness of bacteria, helping them maintain their structural integrity and function in challenging conditions (Obruca et al., 2020). PHAs possess unique properties such as biodegradability, non‐toxicity, biocompatibility; therefore, they are considered as innovative biopolymers that can be used in many novel medical or agricultural applications (Ashori et al., 2019).

The specific details of PHA production can vary depending on the chosen microorganism, carbon source and desired PHA properties. The industrial production of PHA is still scarce, due to the high production costs (Koller & Mukherjee, 2022). Therefore, researchers continue to screen microorganisms that efficiently synthesise PHAs and explore ways to reduce their production costs. Extremophiles are of great interest to scientists because of their numerous applications in the fields of biotechnology (Kaur et al., 2019). The study of extremophiles for PHA production is still relatively in its early stages, but it holds promise for sustainable biopolymers production. Saline environments are widely distributed around the world. They are a source of halophilic bacteria that have a great potential to produce biomolecules such as PHAs. Moreover, in order to develop sustainable and economically viable microbial processes of PHA production, the requirement of the usage of cheap and renewable carbon source is essential. One of industrial feedstocks that can be applied as a substrate for microorganisms, is crude glycerol generated during biodiesel production. Due to the impurities, this residue has limited direct applications and the purification process is too expensive. For this reason, it has become very important to find an alternative method of its utilisation. Moreover, crude glycerol is one of the few waste sources that can be used directly as the carbon source for PHA production (Ray et al., 2016). Therefore, it is considered a promising alternative substrate in reducing production cost (Vicente et al., 2023).

Moreover, based on the concept of next‐generation industrial biotechnology, extremophilic production strains have potential to transform the current PHA production into more competitive bioprocess (Yu et al., 2019). In this context, halophiles are currently considered as candidates for PHA production due to several advantages, including the utilisation of low‐cost carbon sources, reduced risk of contamination and potential for producing tailor‐made PHA compositions with unique properties (Yin et al., 2015). To the best of our knowledge, only in two publications the PHA biosynthesis process using *Halomonas* spp. grown on biodiesel byproducts were investigated. Shrivastav et al. (2010) analysed *Halomonas hydrothermalis* towards poly(3‐hydroxybutyrate) [P(3HB)] production using *Jatropha* biodiesel byproduct. Also, algal biodiesel waste residue was investigated for P(3HB) synthesis by *Halomonas daqingensis* (Dubey & Mishra, 2021). The above‐mentioned reports revealed that *Halomonas* spp. are capable of producing P(3HB) homopolymer. However, none of the study evaluated in details properties of the extracted biopolyesters. Moreover, there is still a lack of studies that showed the potential of *Halomonas* spp. to synthesise PHA copolymers from waste feedstock. In general, P(3HB) homopolymer is stiff and brittle due to its high crystallinity that limits its commercialization. Whereas, poly(3‐hydroxybutyrate‐*co*‐3‐hydrovalerate) copolymer [P(3HB‐*co*‐3HV)] was reported to be more desirable than P(3HB) because their melting point is much lower, and they are less crystalline (Możejko‐Ciesielska & Kiewisz, 2016).

Therefore, the aim of the present study was to evaluate the capability of *Halomonas alkaliantarctica* to produce P(3HB‐*co*‐3HV) copolymer using biodiesel‐derived glycerol without additional precursors. Furthermore, we investigated the effect of the waste substrate concentration on biomass rate and PHA productivity. In addition, the extracted PHA copolymers were comprehensively studied based on their physico‐thermal properties.

## EXPERIMENTAL PROCEDURES

### Microorganism and cultivations

*Halomonas alkaliantarctica* (DSM 15686) was provided by the German Collection of Microorganisms and Cell Culture (Braunschweig, Germany). Seed culture for shake‐flask fermentations was prepared using bacterial stock cultures cryo‐conserved in 20% (*w/v*) of glycerol. Firstly, the bacteria were precultured in Bacto Marine Broth medium (BMB medium) supplemented with 8% NaCl (w/v) at 28°C with shaking (200 rpm) for 16 h before inoculation. The BMB medium contained (per litre): bacto peptone 5 g, bacto yeast extract 1 g, Fe (III) citrate 0.10 g, NaCl 19.45 g, MgCl_2_ 5.90 g, Na_2_SO_4_ 3.24 g, CaCl_2_ 1.80 g, KCl 0.55 g, NaHCO_3_ 0.16 g, KBr 0.08 g, SrCl_2_ 34.0 mg, H_3_BO_3_ 22.0 mg, Na_2_SiO_3_·5 H_2_O 4.0 mg, NaF 2.40 mg, Na_2_HPO_4_ 8.0 mg. Then, shake flasks were inoculated with 10% of the inoculation seed and were subsamples for PHA production performed in 250‐mL Erlenmeyer flasks with 100 mL of fresh BMB medium. All cultivations were carried out using crude glycerol provided by Biofules S.A. Company (Malbork, Poland). The basic parameters of this feedstock were reported previously by Możejko‐Ciesielska and Pokoj (2018): glycerol 85%; NaCl 7%; water 5.5%; organic matter of non‐glycerin 2%, and methanol 0.5%. Effect of the carbon source concentration on PHA production and its properties was determined by adding varying concentrations of biodiesel‐derived glycerol (10, 30, 50, 70 and 80 g/L). The bacterial cells were incubated at 28°C in an orbital shaker at 200 rpm for 72 h.

### Analytical procedures

#### PHA extraction

The cell dry mass (CDM) and PHA concentration was determined at three time‐points (24, 48 and 72 h). *H. alkaliantarctica* cells were centrifuged (11,200 × *g* for 10 min) and after removing the supernatant, the bacterial pellet was lyophilized for 24 h by Lyovac GT2 System (SRK Systemtechnik GmbH, Riedstadt, Germany). PHA extraction was conducted by shaking the freeze‐dried cells in chloroform (purity ≥ 99.8%; Sigma‐Aldrich; USA) for 5 h at 50°C. The obtained mixture was further filtered through No. 1 Whatman filter paper and washed with cold 70% methanol. The collected pellet was allowed to evaporate at the room temperature. PHA content (% of CDM) was determined in the lyophilized biomass and was defined as the percentage of the ratio of PHAs concentration to CDM concentration.

#### Analysis of monomeric composition

The PHA composition was evaluated using Gas Chromatography coupled with Mass Spectrometry (GC–MS QP2010 PLUS, Shimadzu, Japan) as previously reported by Możejko‐Ciesielska and Pokoj (2018). The lyophilized bacterial cells were treated with acidified methanol containing 3% *v/v* H_2_SO_4_ and chloroform. Obtained methyl esters were injected into a BPX70 (25 m × 0.22 mm × 0.25 mm) capillary column (SGE Analytical Science, Victoria, Australia). Helium was used as a carrier gas at 1.38 mL/min. The initial temperature was programmed as 80°C, followed by an increase to 240°C at 10°C/min. The temperature of the ion source was maintained at 240°C. The quantitative analysis was used P(3HB‐co‐3HV) copolymer (Sigma Aldrich, USA) as a standard.

The attenuated total reflectance mode (ATR‐FTIR) of a Fourier Transform Spectrophotometer (FTIR) Nicolet iS10 (ThermoScientific, USA) was used to record infrared spectra of the PHAs samples. The spectral were recorded in the range from 4000 to 650 cm^−1^. Each spectrum analysed was the average of 16 recorded measurements.

#### Analysis of thermal properties

The phase transitions of extracted polymers were determined using a Q200 differential scanning calorimeter (DSC) (TA Instruments, USA). Samples of about 1 mg of the thin PHAs films were first heated to 210°C and kept isothermal for 1 min. to combine several layers of film. Then a cooling run to −30°C was performed at a temperature change rate of 10°C/min. In the last stage, the biopolymers samples were heated to 210°C at a heating rate of 10°C/min. The glass transition temperature (*T*_g_), melting point (*T*_m_), the enthalpy of melting process (Δ*H*_cc_) and degree of crystallinity (*X*_c_) were evaluated from the heating DSC curve. The *X*_c_ values were calculated from the formula:

$$X_{c}=\frac{∆H_{m}-∆H_{\mathrm{cc}}}{∆H_{m100\%}}$$

where Δ*H*_m100%_—change in melting enthalpy of a 100% crystalline sample—109 J/g (Jost et al., 2017).

The thermal resistance of the extracted copolymers was tested using the Q500 thermobalance (TA Instruments, USA). Samples of about 1 mg were evaluated in a nitrogen atmosphere in the temperature range from 25 to 600°C with a temperature change rate of 10°C/min.

## RESULTS AND DISCUSSION

### Effect of crude glycerol concentration on growth and PHA production

Extremophiles have been recently discovered to be capable of utilising renewable feedstocks into high value products (Joulak et al., 2022). The study of halophiles has attracted scientific interest due to their ability to survive in extreme environments and their potential applications in the production of biomolecules. There are no reports that evaluated the potential of *H. alkaliantarctica* to grow and produce PHAs during fermentation process with biodiesel‐derived glycerol. Moreover, any *Halomonas* spp. has not been investigated for production of PHA copolymers using crude glycerol derived from vegetable oil so far. Therefore, the aim of this study was to evaluate whether *H. alkaliantarctica* is capable of converting crude glycerol into PHA copolymers and to characterise them taking into consideration their physico‐chemical properties.

**FIGURE 1:**
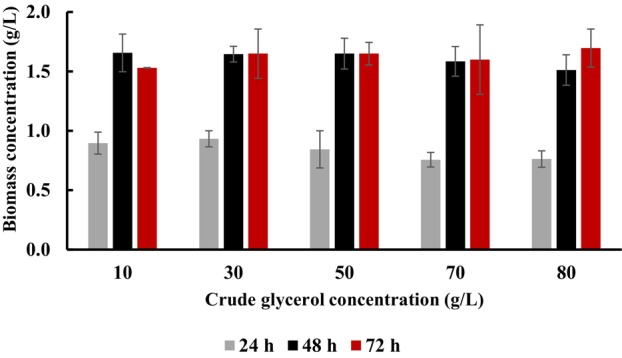
Growth of *Halomonas alkaliantartica* in the medium supplemented with crude glycerol. Mean values are calculated from triplicate measurements.

Our results confirmed that the crude glycerol did not hamper the growth of the analysed bacterial species (Figure 1). We found that CDM values were not dependent on the applying substrate concentration. Shake flasks yielded comparable biomass value at all applied crude glycerol concentrations. Nevertheless, the CDM values were higher at 48 and 72 h in comparison to 24 h of all experimental variants. The biomass concentration reached the maximum value in the cultivation with 80 g/L of crude glycerol in 72 h (1.7 g/L). Lower CDM rates (below 0.5 g/L) were determined by Dubey and Mishra (2021) who cultured *H. daqingensis* in the fermentor supplemented with algal biodiesel waste residue. Lower bacterial cell density was also reported by Shrivastav et al. (2010) in the cultivation of *H. hydrothermalis* with *Jatropha* biodiesel byproduct (about 0.4 g/L).

**FIGURE 2:**
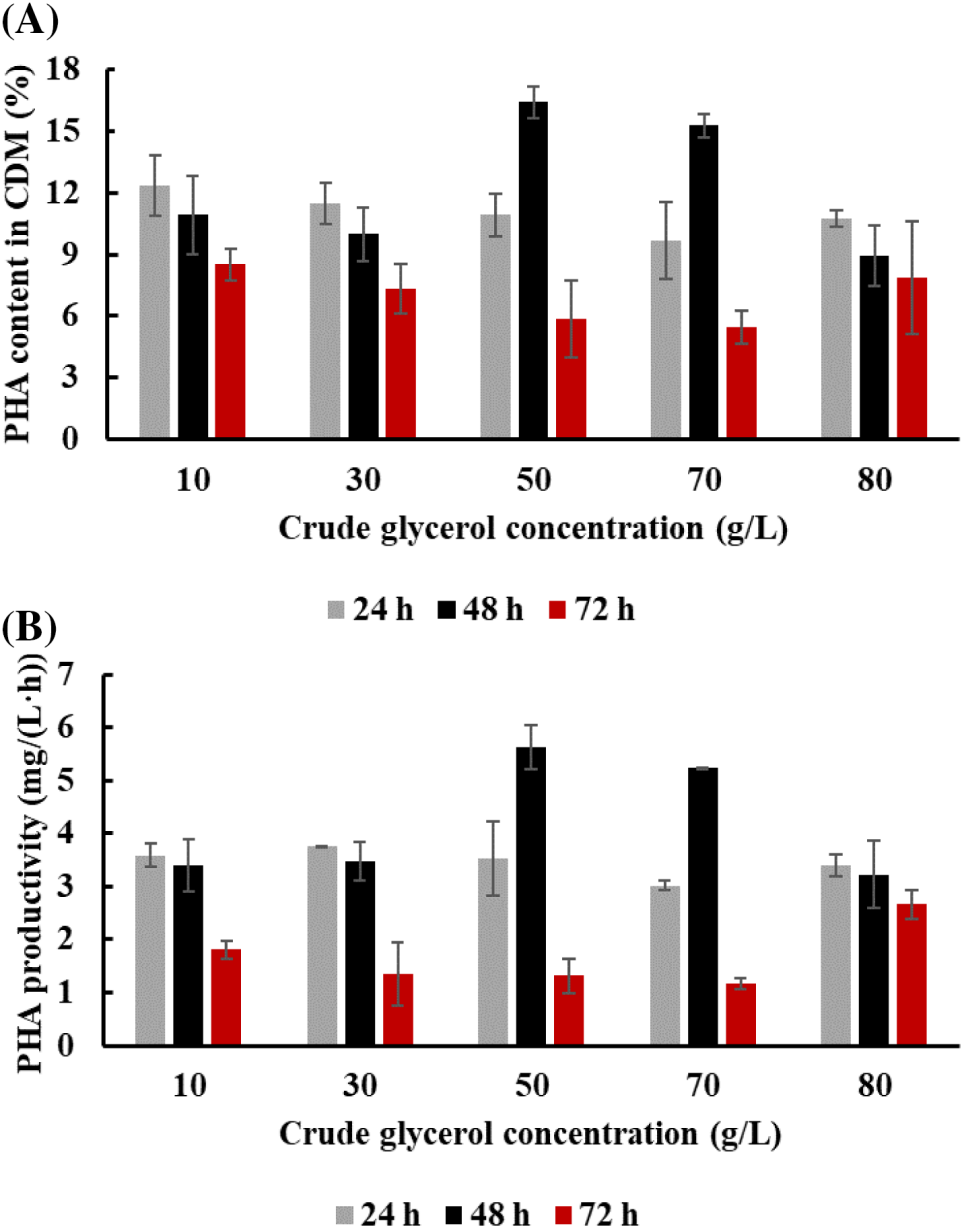
Polyhydroxyalkanoate (PHA) production by *Halomonas alkaliantartica* in the medium supplemented with crude glycerol. (A) PHA content in cell dry mass (CDM), (B) PHA productivity in 24, 48 and 72 h of the cultivations. Mean values are calculated from triplicate measurements.

As shown on Figure 2A,B, *H. alkaliantarctica* was able to produce PHAs in all conducted experiments within 24 h of the cultivation. PHA content was the highest in bacterial cells cultivated in BMB medium supplemented with 50 g/L of biodiesel‐derived glycerol (about 17% of CDM). At higher level of this feedstock, the PHA content in CDM decreased (Figure 2A). However, in all experimental variants the PHA content was higher compared to the values reported in shake‐flasks culture of *H. halophila*, *H. salina* or *H. meridiana* grown on waste frying oil which synthesised only 0.38%, 0.66% and 2.96% of CDM, respectively (Pernicova et al., 2019). Moreover, our results showed that the highest PHA concentration (0.27 g/L) and PHA productivity (5.63 mg/(L·h)) was reached in the cultivation with 50 g/L of crude glycerol in 48 h. We also observed that the further increase of the waste substrate concentration (above 50 g/L) resulted in a decrease of PHAs productivity at all measured time‐points (Figure 2B). However, Liu et al. (2022) suggested that NaCl concentration can play a role in PHA production. The authors showed that pathways involved in salt tolerance can be blocked or weakened in *H. cupida* J9 cells when using glycerol as a feedstock. In a consequence, these bacterial cells can respond to a high salt concentration by increased production of PHA as an effective protectant against salt stress. Also, Kucera et al. (2018) reported that the concentration of NaCl used during cultivation of *H. halophila* influenced PHA productivity. The highest P(3HB) yield was observed in the cultivation supplemented with 60 g/L of NaCl, whereas at lower and higher salt concentration (20, 40, 80 and 100 g/L), the homopolymer productivity decreased. The same observations were made by Rodríguez‐Contreras et al. (2016) in the culture of halophilic bacterium *Bacillus megaterium* uyuni S29. The authors proved that NaCl concentration is an essential factor influencing PHA productivity when employing halophilic bacteria.

### Effect of crude glycerol concentration on PHA composition

The results from gas chromatography coupled with mass spectrometry analysis showed that *H. alkaliantarctica* is capable of producing copolymers contained higher content of 3HB monomer and lower content of 3HV fraction (Table 1). We observed that the repeat units content in the purified PHA copolymers depended on the concentration of biodiesel‐derived glycerol. Moreover, the content of the monomers varied in different cultivation time. The concentration of 3HV fraction was higher in 48 h than in 24 h in all experimental variants. *Halomonas* spp. are known to be able to produce P(3HB) (Dubey & Mishra, 2022; Kawata & Aiba, 2010). Nevertheless, PHA copolymers are of high interest due to their favourable thermomechanical properties compared to PHA homopolymers. Incorporation of 3HV monomer to the polymer chain lowers melting temperature, reduces crystallinity, improves flexibility that are important parameters for their future application (Hammami et al., 2022). Most bacterial species required precursors to produce P(3HB‐*co*‐3HV) such as *Cupriavidus necator* (Grousseau et al., 2014), *Bacillus aryabhattai* (Balakrishna Pillai et al., 2020) or *Halomonas* sp. YLGW01 (Kim et al., 2023). To our knowledge, the analysed *H. alkaliantarctica* is the only halophilic bacterial strain reported to be able to synthesise P(3HB‐*co*‐3HV) copolymers using biodiesel‐derived glycerol without any co‐substrates.

**TABLE 1:** Monomeric composition of extracted PHAs.

Crude glycerol (g/L)	24 h		48 h		72 h	
	3HB (Mol%)	3HV (Mol%)	3HB (Mol%)	3HV (Mol%)	3HB (Mol%)	3HV (Mol%)
10	98.95	1.05	97.23	2.77	97.17	2.83
30	99.03	0.97	97.80	2.20	98.03	1.97
50	98.78	1.22	98.18	1.82	97.77	2.23
70	99.01	0.99	97.93	2.07	98.25	1.75
80	99.00	1.00	98.35	1.65	98.17	1.83

Abbreviations: 3HB, 3‐hydroxybutyrate; 3HV, 3‐hydroxyvalerate; PHA, polyhydroxyalkanoate.

**FIGURE 3:**
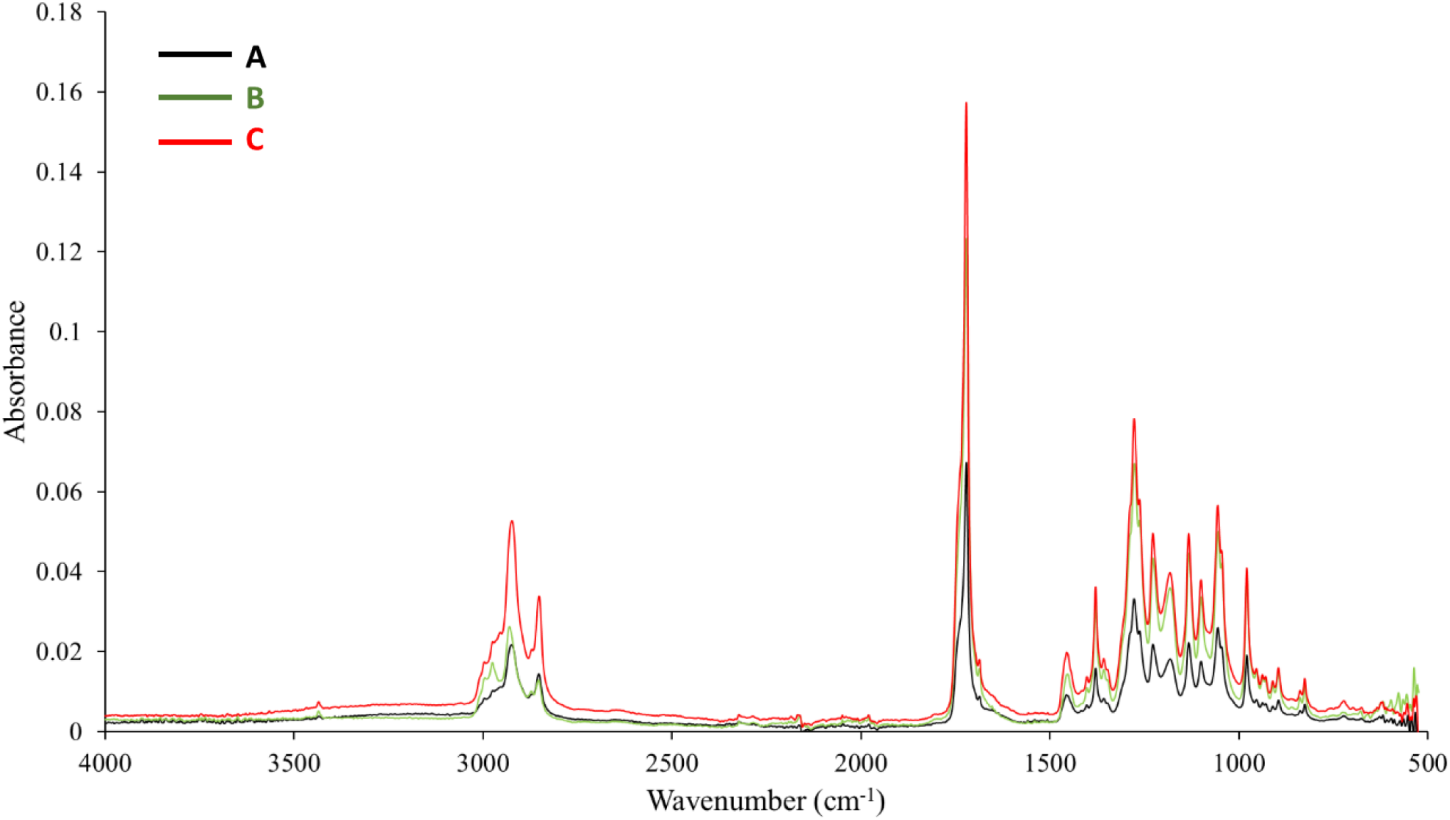
FTIR spectra of selected copolymers extracted from *Halomonas alkaliantartica* cells: (A) P(97.23% HB‐co‐2.77% 3HV) from the cultivation with 10 g/L of crude glycerol; (B) P(98.18% HB‐co‐1.82% 3HV) from the cultivation with 50 g/L of crude glycerol; (C) P(98.35% HB‐co‐1.65% 3HV) from the cultivation with 80 g/L of crude glycerol.

The chemical structure of the extracted biopolymers was also confirmed by Fourier transform infrared spectroscopic spectra (FTIR spectra) (Figure 3). The observed bands were characteristic for P(3HB‐*co*‐3HV) copolymer. Similar signals were reported in earlier studies suggesting the chemical structure of the above‐mentioned PHA copolymer (Volova et al., 2013). The band in the range of 3100–2700 cm^−1^ was associated with the presence of ‐CH_3_ and ‐CH_2_ groups in macromolecules. A single peak band with a maximum of ~1722 cm^−1^ was derived from the C=O carbonyl groups found in macromolecules. Also, Abd El‐malek et al. (2020) showed the strongest band at 1720 cm^−1^ for PHA extracted from *Halomonas pacifica* ASL10 and *Halomonas salifodiane* ASL11. Additionally, our results confirmed that in the spectrum range of 1500–800 cm^−1^, which is the fingerprint region characteristic for PHAs, several bands with numerous peaks are characteristic for the type of the produced copolymers. Peaks with maxima of ~1460, ~1379, ~980, ~900 and ~825 cm^−1^ were associated with the presence of ‐CH_3_ and ‐CH_2_ groups in macromolecules, as well as C‐C bonds. It has been previously proved that a strong vibration at 1379 cm^−1^ is characteristic for PHA (Sathiyanarayanan et al., 2017). Moreover, in the obtained FTIR spectra a series of peaks appeared at ~1278, ~1228, ~1180, ~1132, ~1101 and ~1053 cm^−1^ were associated with bonds in the C‐O‐C region.

### Effect of crude glycerol concentration on the properties of extracted PHAs

Besides chemical structure, the potential applicability of PHAs also depends on its thermal characteristic. Therefore, we investigated the effect of crude glycerol concentration on thermal properties of the extracted co‐polymers (Table 2). Properties studies on the biopolyesters extracted from *Halomonas* spp. cells cultured with biodiesel‐derived glycerol as a sole carbon source have not been reported so far. To characterise comprehensively the PHAs biofilms, the copolymers extracted in 48 h of the cultivations were chosen for further analyses.

**TABLE 2:** Thermal properties of P(3HB‐*co*‐3HV) copolymers extracted from the cultivation of *Halomonas alkaliantarctica* grown on crude glycerol in 48 h.

Crude glycerol (g/L)	PHA composition	Differential scanning calorimetry						Thermogravimetric analysis	
		*T*_g_ [°C]	*T*_cc_ [°C]	Δ*H*_cc_ [J/g]	*T*_m_ [°C]	Δ*H*_m_ [J/g]	*X*_c_ [%]	*T*_d_ [°C]	*T*_max_ [°C]
10	P(97.23% HB‐*co*‐2.77% HV)	−1.5	45.0	49.2	163.6	50.6	1.3	193.9	271.0
30	P(97.80% HB‐*co*‐2.20% HV)	−1.0	48.8	56.4	164.8	62.1	5.2	188.4	285.0
50	P(98.18% HB‐*co*‐1.82% HV)	−0.2	50.3	74.0	165.7	81.7	7.1	212.1	286.1
70	P(97.93% HB‐*co*‐2.07% HV)	0.0	42.7	41.9	165.5	53.7	10.9	213.5	285.9
80	P(98.35% HB‐*co*‐1.65% HV)	0.7	41.3	41.3	165.0	56.1	13.6	213.1	288.3

Abbreviations: P(3HB‐*co*‐3HV), poly(3‐hydroxybutyrate‐*co*‐valerate); *T*_cc_, cold crystallisation temperature; *T*_d_, decomposition temperature; *T*_g_, glass transition temperature; *T*_m_, melting temperature; *T*_max_, maximum decomposition temperature; *X*_c_, degree of crystallinity; Δ*H*_cc_, change in enthalpy of the cold crystallisation process; Δ*H*_m_, change in enthalpy melting process.

**FIGURE 4:**
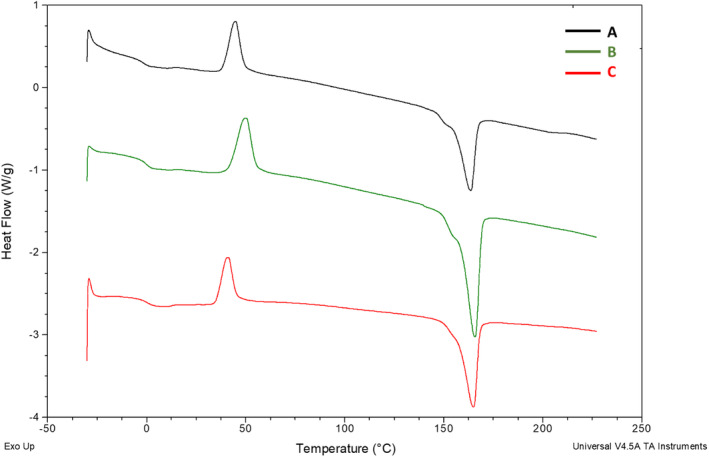
DSC spectra of selected copolymers extracted from *Halomonas alkaliantartica* cells: (A) P(97.23% HB‐co‐2.77% 3HV) from the cultivation with 10 g/L of crude glycerol; (B) P(98.18% HB‐co‐1.82% 3HV) from the cultivation with 50 g/L of crude glycerol; (C) P(98.35% HB‐co‐1.65% 3HV) from the cultivation with 80 g/L of crude glycerol.

For all tested materials, three phase transitions are observed on the DSC curves: glass transition, cold crystallisation and melting (Figure 4). As can be seen in Table 1, the content of 3HV units in individual polymers is very similar. The changes in thermal properties must therefore result from another parameter or material characteristic. It was found that the recorded thermal parameters were strongly influenced by the crude glycerol concentration. We observed that the higher crude glycerol concentration the higher *T*_g_ value. The glass transition temperature (*T*_g_) of the tested materials ranged from −1.5 to 0.7°C. The increase in *T*_g_ was probably due to the change in the degree of crystallinity of the copolymers which also increased with the increase in the concentration of crude glycerol. At a concentration of 10 g/L, the calculated degree of crystallinity was 1.3%, while at a concentration of 80 g/L the degree of crystallinity increased to 13.6%. The melting temperature (*T*_m_) of the analysed biopolymers was also affected by degree of crystallinity and reached about 165°C. This point value is similar to the P(3HB‐*co*‐3HV) isolated from *Halomonas campisalis* cells in the cultivation with bagasse as sole substrate (Kulkarni et al., 2015). Higher *T*_m_ value (178°C) was reported for P(3HB) produced by *Halomonas* sp. SF2003 cultured on agro‐industrial effluents (Lemechko et al., 2019). It could be suggested that the concentration of crude glycerol influences the structure of polymer chains in a way that favours the crystallisation process. The increase in the degree of crystallinity influences the shift in the glass transition and melting temperatures.

**FIGURE 5:**
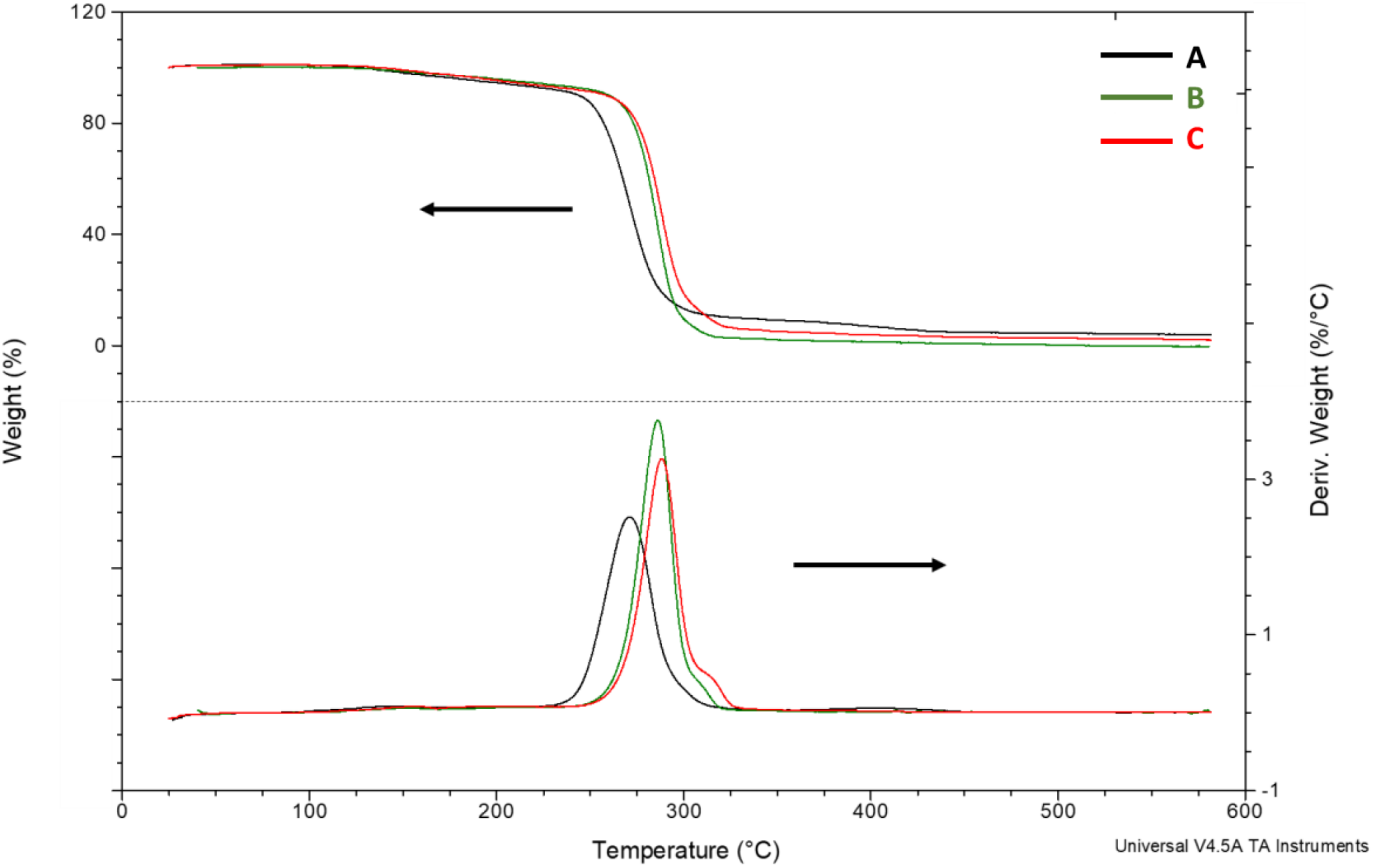
TG and DTG curves of selected copolymers extracted from *Halomonas alkaliantartica* cells: (A) P(97.23% HB‐co‐2.77% 3HV) from the cultivation with 10 g/L of crude glycerol; (B) P(98.18% HB‐co‐1.82% 3HV) from the cultivation with 50 g/L of crude glycerol; (C) P(98.35% HB‐co‐1.65% 3HV) from the cultivation with 80 g/L of crude glycerol.

Furthermore, we observed that the degree of crystallinity increasing with the concentration of crude glycerol influenced on the thermal resistance of the extracted biopolymers (Figure 5). P(3HB‐*co*‐3HV) isolated from the *H. alkaliantarctica* cells grown on higher concentration of crude glycerol were characterised by higher decomposition temperatures (*T*_d_), that is, the parameter adopted as the degree of thermal resistance and representing the temperature of loss of 5% of the sample weight. The highest thermal resistance was detected for the PHA copolymer produced at the highest carbon source concentration (*T*_d_ = 213.1°C). Similar observation was made for maximum degradation temperature (*T*_max_), that is, the parameter determining the temperature at which the thermal degradation process proceeds in the most intensive range. Kavitha et al. (2016) showed that P(3HB) homopolymer produced by *Botryococcus braunii* degraded at 240°C. Our results indicated that the carbon source concentration influenced also on the rate of the degradation process of the extracted copolymers. As the concentration of crude glycerol increased, the *T*_max_ value of analysed PHA copolymers also increased. The *T*_max_ value for PHA biofilm extracted from the cultivation supplemented with 80 g/L reached 288.3°C. Stanley et al. (2020) reported that the maximum degradation for P(3HB) synthesised by *H. venusta* occurred at 323.3°C.

The exploration of halophilic bacteria for PHA production has many benefits such as short fermentation time, lack of pathogenicity and ability to produce this biomolecule using renewable resources (Mitra et al., 2016). Our results proved that *H. alkaliantarctica* is able to utilise crude glycerol for the growth and P(3HB‐*co*‐3HV) production. Also, we found that PHA productivity, monomers content and thermal properties were dependent on the biodiesel‐derived glycerol concentration. In addition, this bacterium produced the scl‐copolymer without any 3HV precursors and make this biotechnological process a valuable strategy and industrially very important. Furthermore, the obtained data suggest that P(3HB‐*co*‐3HV) copolymers have properties that are important for industrial applications. Further work should include metabolic engineering along with genome editing to improve the ability of *H. alkaliantarctica* to metabolise broader range of waste feedstocks. A pressing need also arises to optimise the culture parameters in bioreactor for PHA production at industrial scale.

## AUTHOR CONTRIBUTIONS

**Justyna Możejko‐Ciesielska:** Conceptualization (lead); data curation (lead); formal analysis (supporting); funding acquisition (lead); investigation (lead); methodology (supporting); project administration (lead); resources (lead); supervision (lead); validation (supporting); visualization (supporting); writing – original draft (supporting); writing – review and editing (supporting). **Krzysztof Moraczewski:** Formal analysis (supporting); methodology (supporting); validation (supporting); visualization (supporting); writing – original draft (supporting); writing – review and editing (supporting). **Sylwester Czaplicki:** Methodology (supporting); validation (supporting).

## CONFLICT OF INTEREST STATEMENT

The authors declare no conflicts of interest.

## Data Availability

The data that support the findings of this study are available on request from the corresponding author, Justyna Możejko‐Ciesielska.

## References

[emi413225-bib-0001] Abd El‐malek, F. , Farag, A. , Omar, S. & Khairy, H. (2020) Polyhydroxyalkanoates (PHA) from *Halomonas pacifica* ASL10 and *Halomonas salifodiane* ASL11 isolated from Mariout salt lakes. International Journal of Biological Macromolecules, 161, 1318–1328. Available from: 10.1016/j.ijbiomac.2020.07.258 32755698

[emi413225-bib-0002] Ashori, A. , Jonoobi, M. , Ayrilmis, N. , Shahreki, A. & Fashapoyeh, M.A. (2019) Preparation and characterization of polyhydroxybutyrate‐co‐valerate (PHBV) as green composites using nano reinforcements. International Journal of Biological Macromolecules, 136, 1119–1124. Available from: 10.1016/j.ijbiomac.2019.06.181 31252006

[emi413225-bib-0003] Balakrishna Pillai, A. , Jaya Kumar, A. & Kumarapillai, H. (2020) Biosynthesis of poly(3‐hydroxybutyrate‐*co*‐3‐hydroxyvalerate) (PHBV) in Bacillus aryabhattai and cytotoxicity evaluation of PHBV/poly(ethylene glycol) blends. 3 Biotech, 10, 32. Available from: 10.1007/s13205-019-2017-9 PMC6946779 31988826

[emi413225-bib-0004] Dubey, S. & Mishra, S. (2021) Efficient production of polyhydroxyalkanoate through halophilic bacteria utilizing algal biodiesel waste residue. Frontiers in Bioengineering and Biotechnology, 9, 624859. Available from: 10.3389/fbioe.2021.624859 34604181 PMC8481892

[emi413225-bib-0005] Dubey, S. & Mishra, S. (2022) Natural sea salt based polyhydroxyalkanoate production by wild *Halomonas hydrothermalis* strain. Fuel, 311, 122593. Available from: 10.1016/j.fuel.2021.122593

[emi413225-bib-0006] Grousseau, E. , Blanchet, E. , Déléris, S. , Albuquerque, M.G. , Paul, E. & Uribelarrea, J.L. (2014) Phosphorus limitation strategy to increase propionic acid flux towards 3‐hydroxyvaleric acid monomers in *Cupriavidus necator* . Bioresource Technology, 153, 206–215. Available from: 10.1016/j.biortech.2013.11.072 24365742

[emi413225-bib-0007] Hammami, K. , Souissi, Y. , Souii, A. , Ouertani, A. , El‐Hidri, D. , Jabberi, M. et al. (2022) Extremophilic bacterium *Halomonas desertis* G11 as a cell factory for Poly‐3‐Hydroxybutyrate‐*co*‐3‐Hydroxyvalerate Copolymer's production. Frontiers in Bioengineering and Biotechnology, 10, 878843. Available from: 10.3389/fbioe.2022.878843 35677302 PMC9168272

[emi413225-bib-0008] Jost, V. , Schwarz, M. & Langowski, H.C. (2017) Investigation of the 3‐hydroxyvalerate content and degree of crystallinity of P3HB‐*co*‐3HV cast films using Raman spectroscopy. Polymer (Guildf), 133, 160–170. Available from: 10.1016/j.polymer.2017.11.026

[emi413225-bib-0009] Joulak, I. , Concórdio‐Reis, P. , Torres, C.A.V. , Sevrin, C. , Grandfils, C. , Attia, H. et al. (2022) Sustainable use of agro‐industrial wastes as potential feedstocks for exopolysaccharide production by selected *Halomonas* strains. Environmental Science and Pollution Research International, 29, 22043–22055. Available from: 10.1007/s11356-021-17207-w 34773587

[emi413225-bib-0010] Kaur, A. , Capalash, N. & Sharma, P. (2019) Communication mechanisms in extremophiles: exploring their existence and industrial applications. Microbiological Research, 221, 15–27. Available from: 10.1016/j.micres.2019.01.003 30825938

[emi413225-bib-0011] Kavitha, G. , Kurinjimalar, C. , Sivakumar, K. , Palani, P. & Rengasamy, R. (2016) Biosynthesis, purification and characterization of polyhydroxybutyrate from *Botryococcus braunii* kütz. International Journal of Biological Macromolecules, 89, 700–706. Available from: 10.1016/j.ijbiomac.2016.04.086 27151667

[emi413225-bib-0012] Kawata, Y. & Aiba, S. (2010) Poly(3‐hydroxybutyrate) production by isolated *Halomonas* sp. KM‐1 using waste glycerol. Bioscience, Biotechnology, and Biochemistry, 74, 175–177. Available from: 10.1271/bbb.90459 20057130

[emi413225-bib-0013] Kim, B. , Oh, S.J. , Hwang, J.H. , Kim, H.J. , Shin, N. , Bhatia, S.K. et al. (2023) Polyhydroxybutyrate production from crude glycerol using a highly robust bacterial strain *Halomonas* sp. YLGW01. International Journal of Biological Macromolecules, 236, 123997. Available from: 10.1016/j.ijbiomac.2023.123997 36907298

[emi413225-bib-0014] Koller, M. & Mukherjee, A. (2022) A new wave of industrialization of PHA biopolyesters. Bioengineering (Basel), 9, 74. Available from: 10.3390/bioengineering9020074 35200427 PMC8869736

[emi413225-bib-0015] Kucera, D. , Pernicová, I. , Kovalcik, A. , Koller, M. , Mullerova, L. , Sedlacek, P. et al. (2018) Characterization of the promising poly(3‐hydroxybutyrate) producing halophilic bacterium *Halomonas halophila* . Bioresource Technology, 256, 552–556. Available from: 10.1016/j.biortech.2018.02.062 29478784

[emi413225-bib-0016] Kulkarni, S.O. , Kanekar, P.P. , Jog, J.P. , Sarnaik, S.S. & Nilegaonkar, S.S. (2015) Production of copolymer, poly (hydroxybutyrate‐co‐hydroxyvalerate) by *Halomonas campisalis* MCM B‐1027 using agro‐wastes. International Journal of Biological Macromolecules, 72, 784–789. Available from: 10.1016/j.ijbiomac.2014.09.028 25277119

[emi413225-bib-0017] Lemechko, P. , Le Fellic, M. & Bruzaud, S. (2019) Production of poly(3‐hydroxybutyrate‐*co*‐3‐hydroxyvalerate) using agro‐industrial effluents with tunable proportion of 3‐hydroxyvalerate monomer units. International Journal of Biological Macromolecules, 128, 429–434. Available from: 10.1016/j.ijbiomac.2019.01.170 30707995

[emi413225-bib-0018] Liu, Y. , Zhao, W. , Wang, S. , Huo, K. , Chen, Y. , Guo, H. et al. (2022) Unsterile production of a polyhydroxyalkanoate copolymer by *Halomonas cupida* J9. International Journal of Biological Macromolecules, 223, 240–251. Available from: 10.1016/j.ijbiomac.2022.10.275 36347367

[emi413225-bib-0019] Mitra, R. , Xu, T. , Xiang, H. & Han, J. (2016) Current developments on polyhydroxyalkanoates synthesis by using halophiles as a promising cell factory. Microbial Cell Factories, 19, 86. Available from: 10.1186/s12934-020-01342-z PMC7137286 32264891

[emi413225-bib-0020] Możejko‐Ciesielska, J. & Kiewisz, R. (2016) Bacterial polyhydroxyalkanoates: still fabulous? Microbiological Research, 192, 271–282. Available from: 10.1016/j.micres.2016.07.010 27664746

[emi413225-bib-0021] Możejko‐Ciesielska, J. & Pokoj, T. (2018) Exploring nutrient limitation for polyhydroxyalkanoates synthesis by newly isolated strains of *Aeromonas* sp. using biodiesel‐derived glycerol as a substrate. PeerJ, 6, e5838. Available from: 10.7717/peerj.5838 30370188 PMC6202957

[emi413225-bib-0022] Obruca, S. , Sedlacek, P. , Slaninova, E. , Fritz, I. , Daffert, C. , Meixner, K. et al. (2020) Novel unexpected functions of PHA granules. Applied Microbiology and Biotechnology, 104, 4795–4810. Available from: 10.1007/s00253-020-10568-1 32303817

[emi413225-bib-0023] Pernicova, I. , Kucera, D. , Nebesarova, J. , Kalina, M. , Novackova, I. , Koller, M. et al. (2019) Production of polyhydroxyalkanoates on waste frying oil employing selected *Halomonas* strains. Bioresource Technology, 292, 122028. Available from: 10.1016/j.biortech.2019.122028 31466820

[emi413225-bib-0024] Policastro, G. , Panico, A. & Fabbricino, M. (2021) Improving biological production of poly(3‐hydroxybutyrate‐*co*‐3‐hydroxyvalerate) (PHBV) co‐polymer: a critical review. Reviews in Environmental Science and Biotechnology, 20, 479–513. Available from: 10.1007/s11157-021-09575-z

[emi413225-bib-0025] Ray, S. , Prajapati, V. , Patel, K. & Trivedi, U. (2016) Optimization and characterization of PHA from isolate *Pannonibacter phragmitetus* ERC8 using glycerol waste. International Journal of Biological Macromolecules, 86, 741–749. Available from: 10.1016/j.ijbiomac.2016.02.002 26851207

[emi413225-bib-0026] Rodríguez‐Contreras, A. , Koller, M. , Braunegg, G. & Marqués‐Calvo, M.S. (2016) Poly[(R)‐3‐hydroxybutyrate] production under different salinity conditions by a novel *Bacillus megaterium* strain. New Biotechnology, 33, 73–77. Available from: 10.1016/j.nbt.2015.08.006 26344348

[emi413225-bib-0027] Sathiyanarayanan, G. , Bhatia, S.K. , Song, H.S. , Jeon, J.M. , Kim, J. , Lee, Y.K. et al. (2017) Production and characterization of medium‐chain‐length polyhydroxyalkanoate copolymer from Arctic psychrotrophic bacterium *Pseudomonas* sp. PAMC 28620. International Journal of Biological Macromolecules, 97, 710–720. Available from: 10.1016/j.ijbiomac.2017.01.053 28108411

[emi413225-bib-0028] Shrivastav, A. , Mishra, S.K. , Shethia, B. , Pancha, I. , Jain, D. & Mishra, S. (2010) Isolation of promising bacterial strains from soil and marine environment for polyhydroxyalkanoates (PHAs) production utilizing Jatropha biodiesel byproduct. International Journal of Biological Macromolecules, 47, 283–287. Available from: 10.1016/j.ijbiomac.2010.04.007 20417229

[emi413225-bib-0029] Stanley, A. , Murthy, P.S.K. & Vijayendra, S.V.N. (2020) Characterization of polyhydroxyalkanoate produced by *Halomonas venusta* KT832796. Journal of Polymers and the Environment, 28, 973–983. Available from: 10.1007/s10924-020-01662-6

[emi413225-bib-0030] Vicente, D. , Proença, D.N. & Morais, P.V. (2023) The role of bacterial polyhydroalkanoate (PHA) in a sustainable future: a review on the biological diversity. International Journal of Environmental Research and Public Health, 20(4), 2959. Available from: 10.3390/ijerph20042959 36833658 PMC9957297

[emi413225-bib-0031] Volova, T.G. , Zhila, N.O. , Shishatskaya, E.I. , Mironov, P.V. , Vasil'Ev, A.D. , Sukovatyi, A.G. et al. (2013) The physicochemical properties of polyhydroxyalkanoates with different chemical structures. Polymer Science Series A, 55, 427–437. Available from: 10.1134/S0965545X13070080

[emi413225-bib-0032] Yin, J. , Chen, J.C. , Wu, Q. & Chen, G.Q. (2015) Halophiles, coming stars for industrial biotechnology. Biotechnology Advances, 33, 1433–1442. Available from: 10.1016/j.biotechadv.2014.10.008 25447783

[emi413225-bib-0033] Yu, L.P. , Wu, F.Q. & Chen, G.Q. (2019) Next‐generation industrial biotechnology‐transforming the current industrial biotechnology into competitive processes. Biotechnology Journal, 14, e1800437. Available from: 10.1002/biot.201800437 30927495

